# Awareness, Knowledge, and Attitudes Regarding Basic Life Support Among the General Population in the Al-Majma'ah Region, Saudi Arabia

**DOI:** 10.7759/cureus.48613

**Published:** 2023-11-10

**Authors:** Ahmed H Almutairi, Sultan A Alhassan, Rakan Y Alsuwayyid, Abdulaziz A Alaskar, Fahad S Almutairi, Abdullah F Alsaid, Yousuf Abdulkareem Alharbi, Mohanned A Almazrou, Khalid Fahad K Alotaibi

**Affiliations:** 1 Emergency Medicine, Majmaah University, Al Majma'ah, SAU; 2 College of Medicine, Majmaah University, Al Majma'ah, SAU

**Keywords:** automated external defibrillator, al majmaah, cardiopulmonary resuscitation, emergency, cardiac arrest, basic life support (bls)

## Abstract

Background: Cardiac arrest is a critical medical emergency that can strike individuals of any age or background, often occurring suddenly and unpredictably. The administration of Basic Life Support (BLS) techniques by laypersons in the first few crucial minutes following a cardiac arrest can substantially increase the chances of survival and minimize potential neurological damage. Despite the vital role of BLS in saving lives, there remains a gap in public awareness, knowledge, and attitudes regarding BLS among the general population in many regions worldwide, including Saudi Arabia. In recent years, there has been a growing emphasis on the importance of community-based interventions to enhance cardiac arrest survival rates. Public involvement in the early stages of cardiac arrest management is a key component of the chain of survival, and improving BLS awareness and knowledge among the general population is central to this effort.

Objective: This study aimed to assess the awareness, knowledge, and attitudes with regard to BLS among the general population in the Al-Majma'ah region, Saudi Arabia.

Methods: This is a descriptive cross-sectional study adopted among the population living in the Al-Majma'ah region of Saudi Arabia. The data was collected by a pre-tested and self-administered questionnaire. Data was analyzed by using IBM SPSS Statistics for Windows, Version 26.0 (Released 2019; IBM Corp., Armonk, New York, United States). The questions included information on social demographic information, awareness and knowledge, and attitudes related to BLS.

Results: More than half the participants (n=352; 52.5%) understand that during cardiac arrest, the heart is still beating and pumping blood, but the person is not breathing normally. This is an important understanding for providing proper care during a cardiac arrest situation. On the other hand, the study found that 384 (57.2%) had various reasons for their lack of knowledge about cardiopulmonary resuscitation (CPR). The biggest reason was lack of interest (n=98; 14.6%). This highlights a need for increased awareness and education about the importance of CPR. The findings from the Pearson correlation conducted in this study show that age has a significant influence on the level of awareness and knowledge of cardiac arrest BLS. The p-value obtained for the test was 0.014, indicating that there is a significant relationship between age and awareness and knowledge of BLS. Similarly, the study findings also show that gender has a significant influence on the attitude of cardiac arrest BLS.

Conclusion: The participants had a decent understanding of BLS, particularly regarding concepts like cardiac arrest and the role of automated external defibrillators (AEDs). However, they showed confusion or gaps in awareness, especially concerning the correct initial steps when encountering a collapsed person. Many participants felt uncomfortable performing Hands-Only CPR in a real-life situation due to a lack of knowledge and skills, which acted as a significant barrier to public CPR performance.

## Introduction

Cardiac arrest is a critical medical emergency that can strike individuals of any age or background, often occurring suddenly and unpredictably. It is a leading cause of death worldwide and requires immediate intervention for survival. Out-of-hospital cardiac arrest is experienced by almost 3.8 million people annually [1], and about 60-80% of them die before reaching the hospital [2]. American Heart Association (AHA) defines basic life support (BLS) as the care provided by first responders in case of cardiac or respiratory arrest in order to save someone’s life [3]. The administration of BLS techniques by laypersons in the first few crucial minutes following a cardiac arrest can substantially increase the chances of survival and minimize potential neurological damage [4]. Effective BLS comprises a set of essential skills, including cardiopulmonary resuscitation (CPR), defibrillation, and airway management, which can be administered by bystanders until professional medical help arrives [5]. The morbidity and mortality of those who encounter sudden cardiac arrest have decreased as a result of starting CPR by the witnesses [6-8].

Despite the vital role of BLS in saving lives, there remains a gap in public awareness, knowledge, and attitudes regarding BLS among the general population in many regions worldwide, including Saudi Arabia [9,10]. The prompt initiation of BLS by bystanders is often hampered by a lack of awareness and confidence in performing these life-saving maneuvers [11]. The Al-Majma'ah Region, like many other parts of the world, faces challenges related to BLS awareness and response, which can impact the outcomes of cardiac arrest cases occurring within its communities [12].

Understanding the levels of awareness, knowledge, and attitudes regarding BLS is of paramount importance. Such insight can guide the development of targeted public health campaigns and educational programs aimed at enhancing community preparedness for cardiac and respiratory emergencies [13]. Identifying gaps in BLS knowledge and confidence can help tailor interventions to improve bystander response and ultimately save lives [14].

The significance of this study lies in its potential to shed light on the current status of BLS awareness and knowledge within the Al-Majma'ah Region, Saudi Arabia. Subsequently, these findings can inform the development of evidence-based interventions, educational initiatives, and policy changes designed to increase BLS awareness, competence, and willingness to respond to cardiac arrest incidents within the region [15].

In recent years, there has been a growing emphasis on the importance of community-based interventions to enhance cardiac arrest survival rates [16]. Public involvement in the early stages of cardiac arrest management is a key component of the chain of survival, and improving BLS awareness and knowledge among the general population is central to this effort [17]. Therefore, this study endeavors to contribute to the broader goal of strengthening the community's capacity to respond effectively to cardiac emergencies and, ultimately, to save lives.

## Materials and methods

Study design and setting

This is a descriptive cross-sectional study. A pilot study was done for the questionnaire, the study focuses on the initial validation and refinement of the questionnaire through a small-scale survey. The questionnaire was developed based on a comprehensive review of existing literature and guidelines related to BLS awareness and knowledge [5]. A total of 20 participants were recruited to assess the clarity, comprehensibility, and relevance of the questionnaire items. After conducting the pilot survey, preliminary data were analyzed to identify potential issues and areas for improvement. The findings from this pilot study helped in the finalization of the questionnaire for use in a larger-scale study to assess BLS awareness, knowledge, and attitudes in the target population. Saudi and non-Saudi nationals aged ≥18 years, who were mentally competent, and lived in the Al-Majma'ah region were included. Individuals aged <18 years were excluded. The study was conducted in the Al-Majma'ah region of Saudi Arabia, and approved by the Majmaah University Research Ethics Committee (approval number: MUREC-Sep.11/COM-2023/27-2).

Sample size

The sample size was calculated via the following formula: $n=(z^2 xpq)/d^2$ where n: sample size; Z: standard deviation=1.96; p: prevalence=0.5; q: 1-p; DE: design effect=2; and d: error accepted=0.05. The sample size was calculated to be 384. The participants were selected by non-probability convenience sampling technique.

Data collection and analysis

Data were collected by a pre-tested and self-administered questionnaire. The questions included information on age, gender, and knowledge and attitude regarding BLS. Data were analyzed by using IBM SPSS Statistics for Windows, Version 26.0 (Released 2019; IBM Corp., Armonk, New York, United States). Frequency and percentiles were used for descriptions of categorical variables. Mean and standard deviations were used to describe ongoing variables. The Chi-squared test, t-test, and analysis of variance (ANOVA) were used to evaluate the difference and relationship between variables. The threshold of significance (p-value) was set at 0.05 with a 95% confidence interval (CI).

## Results

Table 1 shows social demographic characteristics of the participants. In total, 671 participants were included in this study. More than half of the participants (n=349; 52%) were female. In terms of age, a total of 283 (42.1%) were aged 25 years and above. The majority (n=405; 60.4%) of the participants were single and 29.4% (n=197) had an income of more than 15,000 Saudi riyals.

**Table 1:** Demographic characteristics of the participants (N=671) Values presented in frequencies (n) and proportion (%) SAR: Saudi Arabia Riyal

Characteristics	n (%)
Gender	
Female	349 (52%)
Male	322 (48%)
Age	
18 to 20 years	187 (27.9%)
21 to 24 years	201 (30%)
More than 25 years	283 (42.1%)
Marital Status	
Divorced	22 (3.3%)
Married	230 (34.3%)
Single	405 (60.4%)
Widowed	14 (2.0%)
Monthly Income	
Less than 5000 SAR	135 (20.1%)
5000 - 9999 SAR	165 (24.6%)
10,000 - 14,999 SAR	174 (25.9%)
More than 15,000 SAR	197 (29.4%)

Table 2 shows the general level of awareness and knowledge of cardiac arrest BLS among the participants. The majority, 352 (52.5%), of the participants knew that during cardiac arrest, the heart of a person is still beating and pumping blood, but the person is not breathing normally. A total of 415 (61.8%) participants generally understood the functions of an AED with 173 (25.8%) of them knowing that it reads the heart rhythm and informs if a shock is needed.

**Table 2:** Level of awareness and knowledge about CPR and BLS among the participants AED: automated external defibrillator; CPR: cardiopulmonary resuscitation; BLS: basic life support Values presented in frequencies (n) and proportion (%).

Variables	n (%)
During cardiac arrest, what happens to a person?	
I don’t know	121 (18.0%)
The heart is still beating and pumping blood, and the person is still alive	45 (6.7%)
The heart stops beating, the person doesn't respond, and the person isn't breathing normally	44 (6.6%)
The heart is still beating, the person isn't breathing normally, and blood stops moving	109 (16.2%)
The heart is still beating and pumping blood, but the person isn't breathing normally	352 (52.5%)
What does an automated external defibrillator (AED) do?	
I don’t know	256 (38.2%)
Reads the heart rhythm and informs if a shock is needed	173 (25.8%)
Automatically phones 911 and calls for help	68 (10.1%)
Informs if a shock is needed and gives the heart rate	133 (19.8%)
Automatically phones 911 and informs if a shock is needed	41 (6.1%)
What is the correct first step when a person has collapsed?	
I don’t know	54 (8.0%)
Tap and shout	85 (12.7%)
Phone 911 and get an AED (if available)	165 (24.6%)
Make sure the scene is safe	104 (15.5%)
Check for breathing	263 (39.2%)
When you do Hands-Only CPR, how many chest compressions should you perform each minute?	
I don’t know	226 (33.7%)
About 50	212 (31.6%)
100 to 120	177 (26.4%)
140 to 150	37 (5.5%)
150 to 200	19 (2.8%)
How deep should you push on the chest of an adult when you do Hands-Only CPR?	
I don’t know	288 (42.9%)
At least 1 inch	81 (12.1%)
At least 2 inches	179 (26.7%)
At least 3 inches	89 (13.3%)
At least 4 inches	34 (5.1%)
Once you shout for help, what are the next steps for providing Hands-Only CPR?	
I don’t know	164 (24.4%)
Phone 911 and get an AED (if available), check for breathing, and begin compressions	154 (23.1%)
Begin compressions, check for breathing, and phone 911 and get an AED (if available)	117 (17.4%)
Phone 911 and get an AED (if available), begin compressions, and check for breathing	76 (11.3%)
Check for breathing, begin compressions, and phone 911, and get an AED (if available)	160 (23.8%)
Would you feel comfortable performing Hands-Only CPR if someone had a cardiac arrest?	
Yes	383 (57.1%)
No	288 (42.9%)
When you perform CPR with breaths on an adult, how many breaths do you give after every 30 compressions?	
I don’t know	214 (31.9%)
2 breaths	287 (42.8%)
4 breaths	96 (14.3%)
6 breaths	54 (8.0%)
8 breaths	20 (3.0%)
What is the correct first step to help a choking adult who stops responding?	
I don’t know	115 (17.1%)
Shout for help	175 (26.1%)
Lay the person on the ground	128 (19.1%)
Phone 911 and get an AED (if available)	138 (20.6%)
Give 30 compressions	115 (17.1%)
What are the signs of a child who is choking?	
I don’t know	62 (9.2%)
The child can speak and play	25 (3.7%)
The child can't speak but can cough	135 (20.1%)
The child can cough and laugh	30 (4.5%)
The child can't cough, speak, or breathe	419 (62.4%)
Out of the following, which source would you say has the biggest attribution to your knowledge about CPR?	
None	384 (57.2%)
University	79 (11.8%)
Internet	53 (7.9%)
Social media	74 (11.0%)
Movies or TV shows	36 (5.4%)
School	11 (1.6%)
Relatives or friends	13 (1.9%)
Others	21 (3.1%)

The correct first step when arriving at a scene where a person has collapsed is to check for breathing, and this was known by 39.2% (n=263) of the participants. A total of 212 (31.6%) participants were aware that above 50 compressions should be performed per minute when Hands-Only CPR is done; of this, 179 (26.7%) participants also indicated that the depth to push on the chest of an adult during Hands-Only CPR should be at least 2 inches. In addition, a total of 160 (23.8%) participants knew that the next steps for providing Hands-Only CPR involved the following: (i) check whether the person is breathing, (ii) begin compressions, and (iii) phone 911 and get an AED (if available).

In terms of comfort level, the majority (n=383; 57.1%) of the participants indicated that they would feel comfortable performing Hands-Only CPR if someone had a cardiac arrest. A total of 287 (42.8%) participants knew that while performing CPR with breaths on an adult, they should give two breaths after every 30 compressions.

Further, 175 (26.1%) indicated that the correct first step to help a choking adult who stops responding is by shouting for help. Nevertheless, 419 (62.4%) participants knew that the signs of a child who is choking are failure to cough, speak, or breathe. Finally, only 287 (42.8%) participants reported that most of their information about CPR came from relatives or friends, school, internet, social media, movies or TV shows, university, and others not specified.

Table 3 shows the general knowledge and attitude among the participants about CPR and its importance in saving lives. A total of 384 (57.2%) participants had varied reasons for the lack of knowledge about CPR. Lack of interest was the main reason (14.6%; n=98). A total of 123 (18.3%) participants indicated a lack of proper knowledge and skills as the primary factor affecting them not performing CPR in public while 575 (85.7%) expressed interest in receiving more CPR training. Additionally, the majority (69.4%; n=466) of participants indicated that the reason for taking more CPR training is to avoid unnecessary death, and 554 (82.6%) participants indicated that they were willing to take free CPR training. Lastly, when asked if CPR training should be mandatory, 78.5% (n=527) of the participants responded that it should be mandatory in every job and at school.

**Table 3:** Attitude regarding CPR and its importance in saving lives CPR: cardiopulmonary resuscitation Data presented in frequencies (n) and proportion (%)

Questions	n (%)
What is the biggest reason for your lack of knowledge about CPR?	
I don’t know	287 (42.8%)
Unknown training location	51 (7.6%)
Lack of time	67 (10.7%)
Cost	21 (3.1%)
Lack of interest	98 (14.6%)
Lack of availability	75 (11.2%)
Others	72 (10.7%)
What is the biggest for a person not being able to perform CPR in public?	
I don’t know	383 (57.1%)
Fear of contagious diseases through mouth-to-mouth breathing	28 (4.2%)
Fear of causing potential harm to the person in need	108 (16.1%)
Fear of legal consequences	16 (2.4%)
Emotional factors	13 (1.9%)
Lack of proper knowledge and skills	123 (18.3%)
Would you be interested in more CPR training in the future?	
Yes	575 (85.7%)
No	96 (14.3%)
What is the reason for more CPR training?	
I don’t know	96 (14.3%)
Heart disease within the family	63 (9.4%)
Wish to avoid unnecessary death	466 (69.4%)
Other reason	46 (6.9%)
Are you willing to take a free CPR course?	
Yes	554 (82.6%)
No	117 (17.4%)
Do you think CPR training should be mandatory?	
Yes, at school	317 (47.2%)
Yes, to obtain a driving license	50 (7.5%)
Yes, in every job	210 (31.3%)
No, CPR training should be optional	94 (14.0%)

Table 4 shows the Pearson correlation between the level of awareness and knowledge of BLS and the social demographic characteristics of the participants. According to the results, age has a significant influence on the level of awareness and knowledge of cardiac arrest BLS (p=0.014).

**Table 4:** Correlation between level of awareness and knowledge of BLS in cardiac arrest and social demographic characteristics. p<0.05 is considered significant BLS: basic life support

Demographic Characteristics		p-value
Gender	Pearson Correlation	0.036
	Sig. (2-tailed)	0.351
Age	Pearson Correlation	0.095
	Sig. (2-tailed)	0.014
Marital Status	Pearson Correlation	0.000
	Sig. (2-tailed)	0.997
Monthly oncome	Pearson Correlation	0.046
	Sig. (2-tailed)	0.234

Table 5 shows the Pearson correlation between the attitude toward cardiac arrest BLS and the social demographic characteristics of the participants. According to the results, the gender of the participants had a significant influence on the attitude toward cardiac arrest BLS (p=0.000).

**Table 5:** Correlation between attitude toward BLS in cardiac arrest and social demographic characteristics (N=671) p<0.05 is significant BLS: basic life support

Demographic Characteristics		p-value
Gender	Pearson Correlation	0.153
	Sig. (2-tailed)	0.000
Age	Pearson Correlation	0.007
	Sig. (2-tailed)	0.866
Marital Status	Pearson Correlation	0.024
	Sig. (2-tailed)	0.538
Monthly income	Pearson Correlation	0.015
	Sig. (2-tailed)	0.698

## Discussion

A total of 352 (52.5%) participants in the current study understand that during cardiac arrest, the heart is still beating and pumping blood, but the person is not breathing normally. This is an important understanding for providing proper care during a cardiac arrest situation. Furthermore, 61.8% (n=415) of participants generally understood the functions of an AED, with 25.8% (n=173) correctly identifying that it reads the heart rhythm and determines if a shock is needed. This knowledge is crucial for using an AED effectively in emergency situations. In terms of the correct first step when arriving at a scene where a person has collapsed, 39.2% (n=263) of participants knew that it is to check for breathing. This demonstrates a basic understanding of the initial actions to take when encountering a person in distress.

Jarrah et al. found that 14.1% of the participants had previously received some kind of BLS training, and their overall knowledge about BLS was moderate. Furthermore, they highlighted the need for increased public education and training in BLS [18]. In the current study, the number of people previously trained for BLS has not been found but, similar to the study by Jarrah et al., it highlighted the need for increased training in BLS as over 80% of the study participants felt it should be mandatory either at school, jobs, or for driving licenses, and over 80% are willing to take a free CPR training course.

Regarding Hands-Only CPR, about 212 (31.6%) participants knew that more than 50 compressions should be performed per minute. This information is essential for providing effective CPR and maintaining blood flow to vital organs. Additionally, a total of 179 (26.7%) of participants correctly indicated that the depth to push on the chest during Hands-Only CPR should be at least 2 inches. This shows a basic understanding of the proper technique for performing chest compressions. When asked about the next steps for providing Hands-Only CPR, 160 (23.8%) participants knew that they should check for breathing, begin compressions, and call 911 and retrieve an AED if available, which shows that they understand the importance of a step-by-step approach in providing emergency care. In terms of comfort level, the majority (57.1%, n=383) of participants indicated that they would feel comfortable performing Hands-Only CPR if someone had a cardiac arrest. This is a positive finding. Moving on to CPR with breaths on an adult, 287 (42.8%) participants indicated that they would give two breaths after every 30 compressions, which shows an understanding of the proper ratio of chest compressions to rescue breaths in traditional CPR. When it comes to helping a choking adult who stops responding, about 175 (26.1%) of participants correctly identified that the first step is to shout for help. This demonstrates a basic understanding of the initial actions to take in a choking emergency. Regarding signs of a choking child, 62.8% (n=421) of participants correctly identified failure to cough, speak, or breathe as indicators of choking. This knowledge is crucial for recognizing and responding appropriately in a choking situation involving a child. These findings concur with Ghanem et al.'s study finding which found that the overall awareness of BLS among the participants was low, with only 48.7% having adequate knowledge [19]. Their study highlighted the need for further educational interventions to improve BLS awareness and skills among professionals in the medical field in Egypt.

On the other hand, the study found that 352 (57.2%) participants had various reasons for their lack of knowledge about CPR. The biggest reason was lack of interest, cited by 98 (14.6%) participants. This highlights a need for increased awareness and education about the importance of CPR. Interestingly, 18.3% (n=123) of participants indicated that a lack of proper knowledge and skills was the primary factor preventing them from performing CPR in public. This suggests that many people may feel unsure of their abilities and are hesitant to try CPR without proper training. This reinforces the importance of providing accessible and comprehensive CPR training to the public. It is encouraging to note that most participants, 85.7% (n=575), expressed interest in receiving more CPR training in the future. This shows a willingness to learn and improve their skills, which can be instrumental in saving lives in emergency situations. When asked about the reasons for taking more CPR training, the majority (69.4%, n=466) stated that it is to avoid unnecessary death. This indicates a strong understanding of the life-saving potential of CPR and the motivation to make a difference in emergency situations. Interestingly, a significant majority (82.6%, n=554) of participants indicated that they are willing to take free CPR training. This highlights the importance of removing financial barriers to CPR training and making it accessible to everyone. Lastly, when asked about whether CPR training should be mandatory, 527 (78.5%) believed that it should be mandatory in every job and at school. This reflects a recognition of the importance of CPR knowledge and skills in various settings and the need for widespread training to ensure that everyone is prepared to respond to cardiac arrest emergencies [20,21]. These findings are consistent with Özbilgin et al.'s study in Türkiye, which revealed that most participants had heard about CPR, but their knowledge of correct CPR techniques was insufficient [22].

There is a significant relationship between age and awareness and knowledge of BLS (p-value=0.014. Similarly, gender has a significant influence on the attitude toward BLS (p-value=0.000). These findings go hand in hand with Ghrayeb and his colleagues’ study findings, which showed a lack of knowledge and inadequate training in BLS among teachers, and found varied levels of knowledge and attitude regarding BLS among the teachers based on gender and age with the middle-aged female group having better knowledge [23].

Limitations

Limitations of the study include the small sample size in a specific geographic location, which may limit the generalizability of the findings to a larger population in Saudi Arabia. Additionally, the study relied on self-reported data, which may be subject to recall bias or social desirability bias. The study also did not assess the participants' actual skills in performing CPR, but only their knowledge and attitudes towards CPR.

Recommendations

Based on the findings of this study, a number of recommendations can be made to improve the readiness of the general public to provide BLS during cardiac arrest. Targeted education and awareness programs should be developed to improve overall knowledge and understanding of BLS concepts and procedures, with a specific focus on the correct first steps to take when arriving at a scene where someone has collapsed. Efforts should be made to address the barriers identified, such as lack of interest and fear of legal consequences, by highlighting the importance of BLS and addressing misconceptions. Mandatory CPR training in workplaces should be implemented to ensure that employees are equipped with the necessary skills to respond to cardiac arrest emergencies. Tailored interventions should be designed to target specific demographic groups, such as younger individuals and males, who were found to have lower levels of awareness and knowledge about BLS. Finally, further research should be conducted to explore the reasons behind the lack of interest in receiving CPR training and to identify strategies to motivate individuals to participate in training courses.

## Conclusions

This study provides insight into the social demographic characteristics, knowledge, attitudes, and barriers related to BLS during cardiac arrest among the participants. Most participants were female, aged 25 years and above, and single. Overall, the participants demonstrated some level of awareness and knowledge about BLS, particularly regarding concepts like cardiac arrest and the role of AEDs. However, there were areas of confusion or lack of awareness, such as the correct first step when arriving at a scene where someone has collapsed. Participants reported feeling relatively uncomfortable performing Hands-Only CPR in a real-life situation. Lack of proper knowledge and skills were identified as significant barriers to performing CPR in public. Most participants expressed interest in receiving further CPR training in the future and were willing to take a free CPR course. They recognized the importance of mandatory CPR training in workplaces and schools. Age and gender were found to have a significant influence on awareness, knowledge, and attitudes with regard to BLS.
